# A binding-block ion selective mechanism revealed by a Na/K selective channel

**DOI:** 10.1007/s13238-017-0465-8

**Published:** 2017-09-18

**Authors:** Jie Yu, Bing Zhang, Yixiao Zhang, Cong-qiao Xu, Wei Zhuo, Jingpeng Ge, Jun Li, Ning Gao, Yang Li, Maojun Yang

**Affiliations:** 1 Ministry of Education Key Laboratory of Protein Science, School of Life Sciences, Tsinghua-Peking Joint Center for Life Sciences, Beijing Advanced Innovation Center for Structural Biology, Tsinghua University, Beijing, 100084 China; 2 Key Laboratory of Receptor Research, Shanghai Institute of Materia Medica, Chinese Academy of Sciences, Shanghai, 201203 China; 3 Department of Chemistry and Key Laboratory of Organic Optoelectronics and Molecular Engineering of the Ministry of Education, Tsinghua University, Beijing, 100084 China; 4 University of Chinese Academy of Sciences, Beijing, 100049 China; 5 Anesthesiology, Shanghai First Maternity and Infant Hospital, Tongji University school of Medicine, Shanghai, 201203 China

**Keywords:** cryo-EM, MscS, Na^+^/K^+^ selective channel

## Abstract

Mechanosensitive (MS) channels are extensively studied membrane protein for maintaining intracellular homeostasis through translocating solutes and ions across the membrane, but its mechanisms of channel gating and ion selectivity are largely unknown. Here, we identified the YnaI channel as the Na^+^/K^+^ cation-selective MS channel and solved its structure at 3.8 Å by cryo-EM single-particle method. YnaI exhibits low conductance among the family of MS channels in *E*. *coli*, and shares a similar overall heptamer structure fold with previously studied MscS channels. By combining structural based mutagenesis, quantum mechanical and electrophysiological characterizations, we revealed that ion selective filter formed by seven hydrophobic methionine (YnaI^Met158^) in the transmembrane pore determined ion selectivity, and both ion selectivity and gating of YnaI channel were affected by accompanying anions in solution. Further quantum simulation and functional validation support that the distinct binding energies with various anions to YnaI^Met158^ facilitate Na^+^/K^+^ pass through, which was defined as binding-block mechanism. Our structural and functional studies provided a new perspective for understanding the mechanism of how MS channels select ions driven by mechanical force.

**Electronic supplementary material:**

The online version of this article (doi:10.1007/s13238-017-0465-8) contains supplementary material, which is available to authorized users.

## Introduction

Substantial progresses have been achieved in addressing the fundamental question of gating and ion selectivity in ion channels studies. The molecular mechanisms of how the ions are selected have been widely explored, such as the cation-selective potassium (Brohawn et al., 2012; Fox and Richards, 1982; Hite et al., 2015; Kawate et al., 2009; Valiyaveetil et al., 2006; Zhou et al., 2001), sodium (Baconguis et al., 2014; McCusker et al., 2012; Payandeh et al., 2012; Payandeh et al., 2011; Zhang et al., 2012a), or calcium channels (Hou et al., 2012; Jiang et al., 2002; Van Petegem et al., 2004; Wagenknecht et al., 1989; Wu et al., 2015), anion-selective channels (Kane Dickson et al., 2014; Zhang et al., 2012b), and mechanosensitive (MS) channels (Bass et al., 2002; Bottcher et al., 2015; Chang et al., 1998; Ge et al., 2015; Perozo et al., 2002; Wang et al., 2008; Zhang et al., 2012b). Mechanosensitive channel of small conductance (MscS) has an indispensable role in the protection of bacterial cells when the cells experience a transfer from a high osmolarity medium to a low osmolarity environment. MscS have been investigated on structural and functional levels and displayed different ion selectivities. Previous studies demonstrated that MscS from *E*. *coli (Ec*MscS) had a slight anion preference (P_Cl_/P_K_ ~1.5–3), whereas MscS from *Thermoanaerobacter tengcongensis* (*Tt*MscS) had a strong anion preference (P_Cl_/P_K_ ~39). The underlying molecular mechanism for anion-selective MscS has been elucidated deeply, but for MscS with cation selectivities remains elusive.

In the present study, we characterize a Na^+^/K^+^ cation-selective MS channel, YnaI, which exhibits small conductance among the family of MS channels in *E*. *coli* (Bottcher et al., 2015). We solved the structure of YnaI at 3.8 Å resolution by single-particle cryo-EM. Guided by structural information, mutant channels were constructed and reconstituted into liposomes for electrophysiological characterization. Functional results indicated that the cation selectivity of YnaI channel was affected by accompanying anions in solution. Our data revealed that YnaI^Met158^ in the transmembrane pore was instrumental in determination of ion selectivity. Further simulation and mutagenesis validation supported that seven methionine residues formed a circle and bound anions with different binding energy, leading to elegant modulation of further passing of cations. Our study defines a novel role of the transmembrane region in ion selection of a Na^+^/K^+^-selective MscS channel and provides a new venue for understanding the selectivity and gating mechanism of ion channels.

## Results

### YnaI is a Na^+^/K^+^ selective mechanosensitive channel

**Figure 1:**
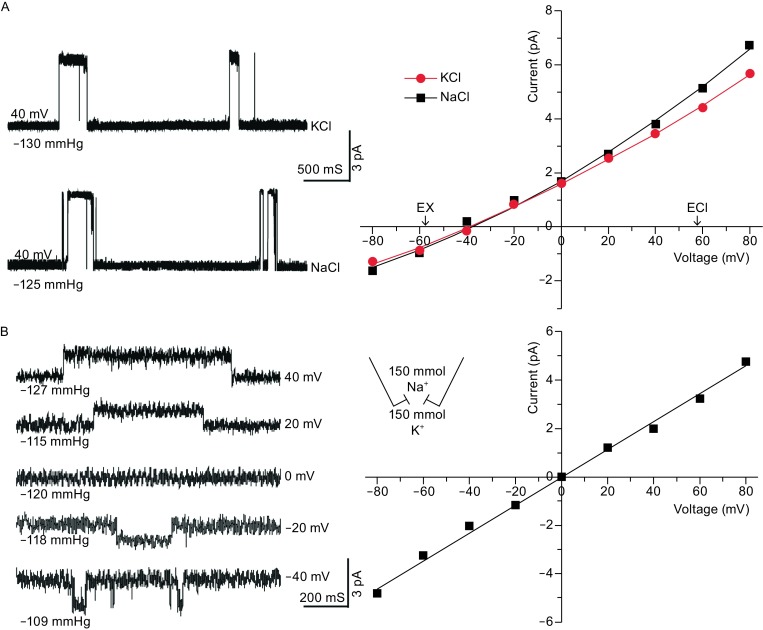
**YnaI is a Na** ^**+**^ **/K** ^**+**^ **selective mechanosensitive channel**. (A) Left: single-channel traces were recorded by patch-clamp system from giant liposomes in KCl solution (upper) or NaCl solution (lower) at +40 mV. YnaI mutants were recorded in only KCl solution by the same method. The numbers under the single-channel traces represent the negative pressure applied to the patch during the event. Right: I–V curves for YnaI channel at a 10:1 salt gradient (150 mmol/L/15 mmol/L, KCl or NaCl); the reversal potentials of an ideal anion or cation-selective channel with Erev = +58 mV or −58 mV according to the Nernst equation are indicated. The reversal potentials for YnaI at KCl or NaCl solution are −39.2 ± 1.1 mV (*n* = 5, mean ± SE) and −40 ± 1.0 mV (*n* = 6, mean ± SE), respectively. (B) Left: single-channel traces of YnaI at different voltages (0 mV, ±20 mV, ±40 mV), with recording solutions filled by 150 mmol/L NaCl in the pipette and 150 mmol/L KCl in bath solutions, respectively. Right: I–V curve for YnaI channel at the condition that had described in left panel. The reversal potential was changed to 0 mV under 150 mmol/L NaCl/150 mmol/L KCl condition

Sodium/potassium transport is essential for cell growth due to its roles in generating turgor pressure and regulating cytoplasmic pH (Brohawn et al., 2012; Fox and Richards, 1982; Hite et al., 2015; Kawate et al., 2009; Valiyaveetil et al., 2006; Zhou et al., 2001). We verified electrophysiological activities of YnaI in patch-clamp system using recombinant proteins and *in vitro* reconstituted giant liposomes. YnaI in asymmetric KCl solutions (150 mmol/L/15 mmol/L) exhibited a mean reversal potential of −39.2 ± 0.5 mV (*n* = 5 patches), corresponding to a cation-to-anion (P_K_:P_Cl_) permeability ratio of about 9:1. Similar results also obtained in NaCl solutions (Fig. 1A). Interestingly, YnaI could not distinguish Na^+^/K^+^ in the symmetric K^+^/Na^+^ solutions (150 mmol/L KCl/150 mmol/L NaCl) (*n* = 5 patches) (Fig. 1B). These data suggested that YnaI was a Na^+^/K^+^- selective MS channel.

### YnaI has a similar overall structure fold with *Tt*MscS and *Ec*MscS

**Figure 2:**
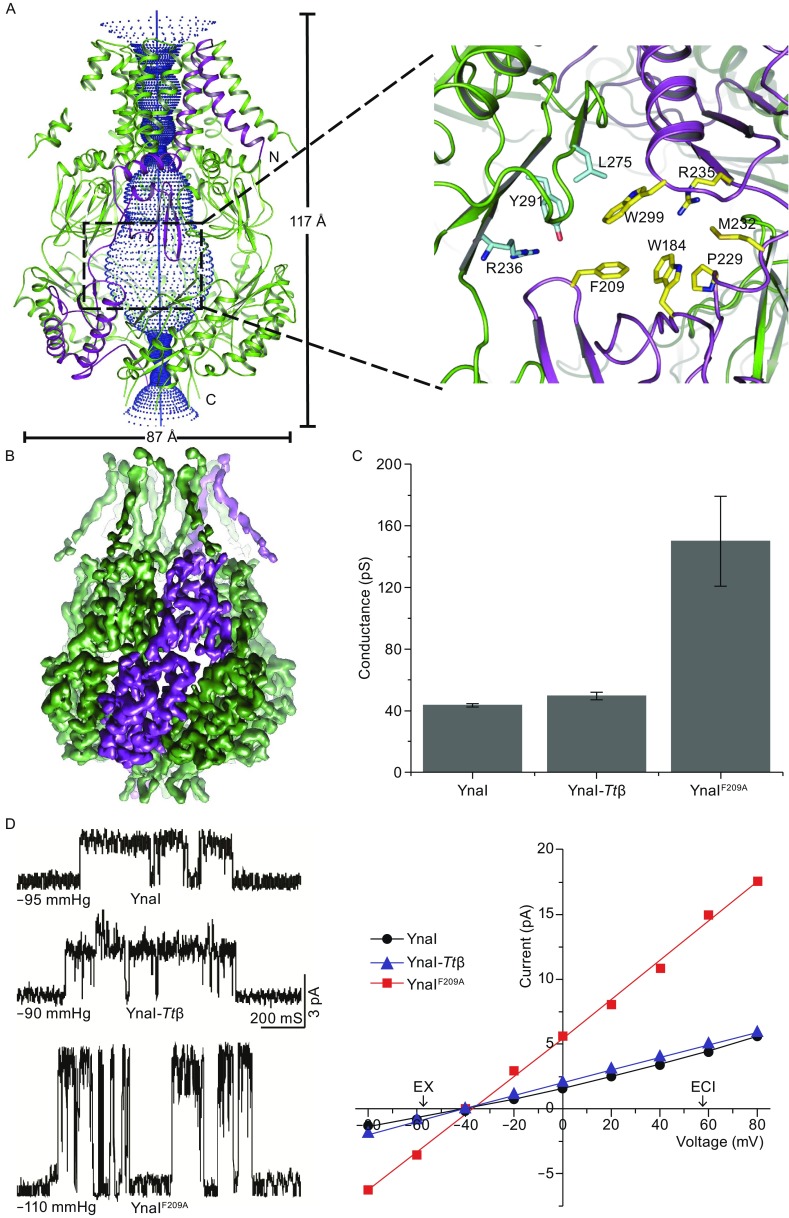
**The cytoplasmic equatorial portals of YnaI contribute to ion conductance**. (A) Right: overall structure of YnaI homoheptamer. One protomer is colored in purple, and the others are colored in green. The channel passage is shown in blue dots along a blue axis. Left: ribbon diagram of close views of one of the seven portals in YnaI. Residues lining the portals are shown in yellow and cyan sticks. (B) Cryo-EM density map of YnaI, with one of the seven promoters highlighted in dark purple. (C) YnaI^F209A^ mutant showed a higher conductance comparing with wide-type YnaI and YnaI mutant substituted with the *Tt*MscS β-barrel region (YnaI-*Tt*β) (*n* = 4, mean ± SE). (D) Left: single-channel traces of YnaI, YnaI-*Tt*β and YnaI^F209A^ mutant were recorded at +40 mV. Right: I–V curves for YnaI, YnaI-*Tt*β, and YnaI^F209A^ mutant. Both YnaI-*Tt*β and YnaI^F209A^ mutants shared a similar reversal potential with YnaI. YnaI^F209A^ displayed an obviously higher conductance

To understand the molecular mechanism of the Na^+^/K^+^-selective characterization of YnaI, we solved its structure at an overall resolution of 3.8 Å (3.6 Å at the cytoplasmic domain) by cryo-EM single-particle method (Figs. 2B, S1, and S2). Similar to previously reported MscS structures (Bass et al., 2002; Wang et al., 2008; Zhang et al., 2012b), YnaI comprises seven YnaI promoters bearing the MscS channel fold and forming a homoheptamer (Fig. 2A). The heptamer extends ~117 Å parallel to the sevenfold axis, with ~87 Å in width in the perpendicular direction (Fig. S3). Secondary structure analysis predicts that YnaI contains five TM helices (Fig. S4), while we only identified two transmembrane helices (TMH4 and TMH5) in each YnaI protomer, which may be due to the flexibility of the other three TMHs. TMH5 lines the channel pore (Fig. S3).

Consistent with other MscS channels, YnaI contains a large cytoplasmic domain and comprises the middle β-domain, α/β domain, and a C-terminal extension forming an interior chamber of about 30 Å in diameter (Fig. S3) (Bass et al., 2002; Wang et al., 2008). The isolated C-terminus of β10 in each monomer forms a seven-stranded parallel β-barrel (residues 327–343), leading to the formation of a potential channel pore (referred to as the β-barrel pore hereafter). The structure of YnaI closely resembles that of the closed conformation of *Tt*MscS and *Ec*MscS with root-mean-square deviations (rmsd) of 3.05 Å and 2.3 Å over 223 Cα atoms of the overall structures, respectively (Fig. S3). These structural observations suggest that YnaI structure was resolved at a non-conducting state.

### YnaI^M158^ is identified as a main determinant for ion selectivity

**Figure 3:**
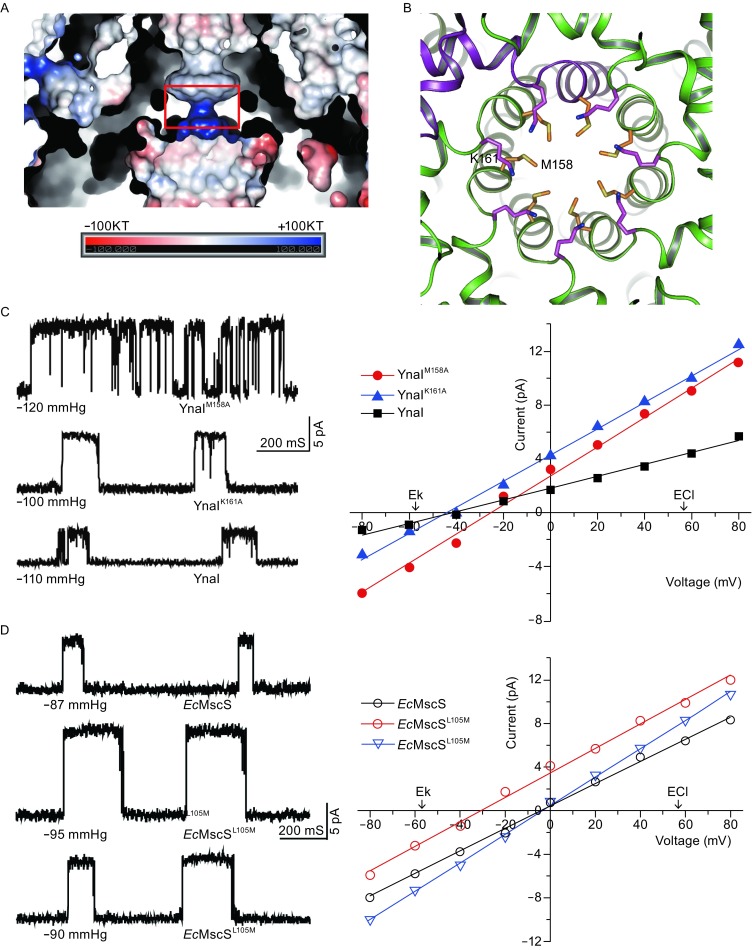
**YnaI** ^**M158**^ **located at transmembrane region determines the cation selectivity**. (A) Electrostatic potentials around the transmembrane pore inner surface of YnaI. Narrowest region where M158 and K161 located is indicated by red square. (B) Ribbon diagram of TM helices surrounding restriction site viewed along membrane bilayer from extracellular side. M158 and K161 are shown in yellow and purple sticks, respectively. (C) Mutation in the transmembrane region decreased the cation selectivity of YnaI. Left: single-channel traces of YnaI, YnaI^M158A^, and YnaI^K161A^ were recorded at +40 mV. Right: I–V curves for YnaI, YnaI^M158A^, and YnaI^K161A^. The reversal potential of M158A shifted right (−26 ± 1.5 mV, *n* = 3, mean ± SE), representing an attenuation of cation selectivity. (D) Mutation of the key residues in gating ion endowed *Ec*MscS channel cation selectivity. Left: single-channel traces of *Ec*MscS, *Ec*MscS^L105M^, and *Ec*MscS^L109M^ mutants were recorded at +40 mV. Right: I–V curves for *Ec*MscS, *Ec*MscS^L105M^, and *Ec*MscS^L109M^ mutants. The reversal potential of *Ec*MscS^L105M^ shifted right (−31.1 ± 0.6 mV, *n* = 6, mean ± SE), representing an increased cation selectivity

Previous studies suggested that the seven portals in the cytoplasmic domain (Bass et al., 2002; Wang et al., 2008) and the β-barrel region (Zhang et al., 2012b) were involved in the ion selectivities. In order to determine whether these regions are also in charge of the ion selectivity in YnaI channel, we purified the mutant proteins with predicted enlarged portals (YnaI^F209A^) (Fig. 2A) or a chimera YnaI-*Tt*β with the β-barrel replaced by the β-barrel region from the anion-selective *Tt*MscS (Zhang et al., 2012b) and recorded their electrophysiological activities (Fig. 2C and 2D). Enlarged portals increased the ion conductance current as expected (about 4 times), while replacement of the β-barrel only resulted in a slight increase. Surprisingly, no changes occur in the ion selectivities of both mutants, which suggests that the ion selective pore may lie in the TM region (Fig. 3A). Indeed, YnaI^M158A^ in the TM region alters the mean reversal potential from −39.2 ± 0.5 mV to −20 ± 0.5 mV, corresponding to decreased cation-to-anion (P_K_:P_Cl_) permeability ratio from 9:1 to 3:1 (Fig. 3A–C). Nevertheless, the YnaI^K161A^ mutant only results in the increase of ion conductance but no variance in ion selectivity (Fig. 3C). More interestingly, mutation of the key residues that gate the non-selective *Ec*MscS is consistent with our findings that the *Ec*MscS^L105M^ (*Ec*MscS^L105^ corresponds to the YnaI^M158^) obtains cation selectivity compared with the wild type (Bass et al., 2002; Rasmussen et al., 2015), while the *Ec*MscS^L109M^ (*Ec*MscS^L109^ corresponds to the YnaI^K161^) mutation has no effect on the ion selectivity (Figs. 3D and S5). Collectively, these data provide strong evidence that a circular pore (SMC, seven-methionine circle) formed by the seven YnaI^M158^ residues could be an important structural determinant for ion selectivity in YnaI.

### YnaI^M158^ binding various anion with distinct binding energies facilitates Na^+^/K^+^ pass through

**Figure 4:**
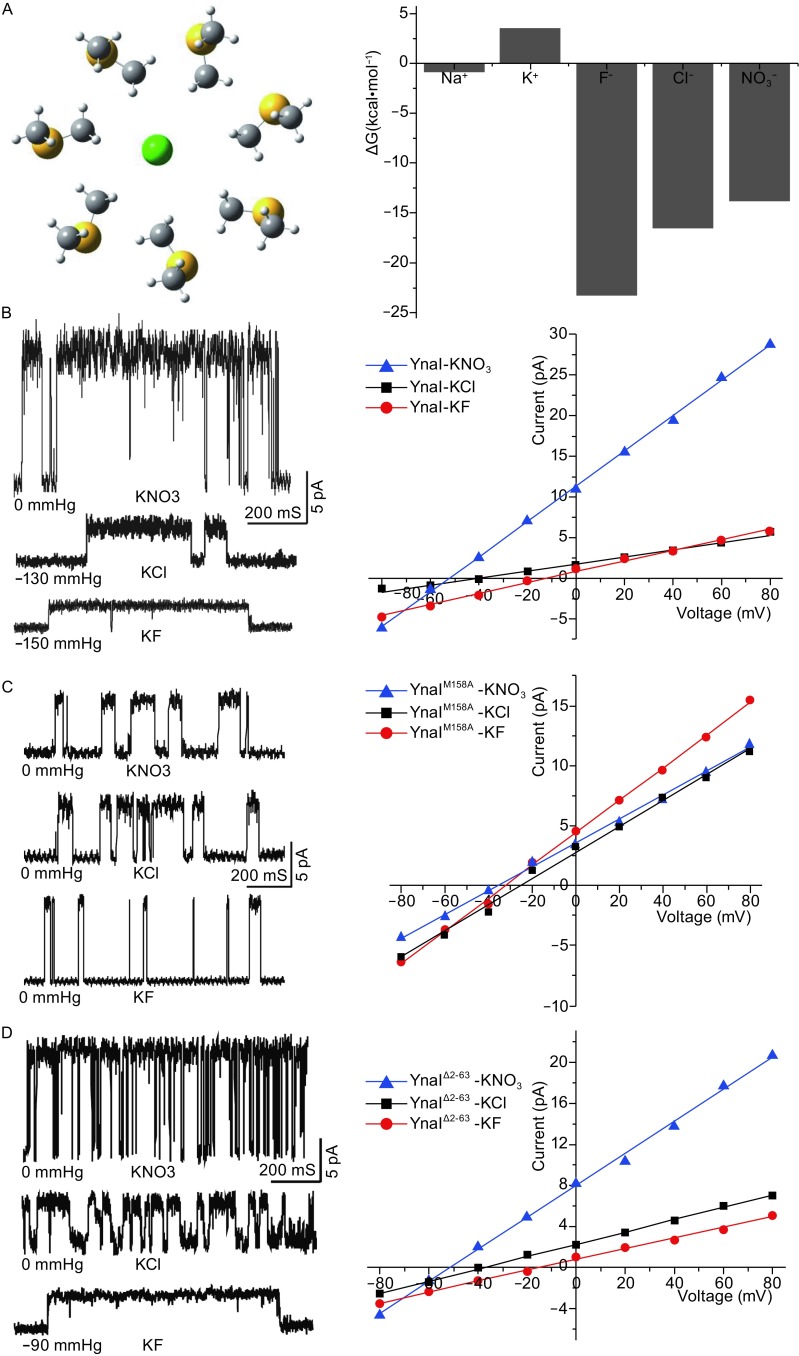
**Different anions affect the ion selectivity and transmittance of YnaI**. (A) Optimized structures of (H_3_CSCH_3_)_7_ and Cl@(H_3_CSCH_3_)_7_, where van der Waals radii were used in the later to illustrate the size of the cavity. Relative binding energies for A + (H_3_CSCH_3_)_7_ → A@(H_3_CSCH_3_)_7_ (A = Na^+^, K^+^, F^−^, Cl^−^, Br^−^). (B) Left: single-channel traces of YnaI were recorded at +40 mV in different asymmetric potassium salt solutions. The required negative pressures for opening the channel were different in those solutions. In KF, the required negative pressure was −150 ± 13 mmHg (*n* = 4, mean ± SE). In KCl, the required negative pressure was −130 ± 11 mmHg (*n* = 5, mean ± SE). In KNO_3_, YnaI channel opened spontaneously without pressure applied (*n* = 4). Right: I–V curves for YnaI at different asymmetric potassium salt solutions. The reversal potentials of YnaI varied in different solutions. In KF, KCl and KNO_3_, the reversal potentials were −19.2 ± 1.6 (*n* = 4, mean ± SE), −39.2 ± 0.5 (*n* = 5, mean ± SE), −52.3 ± 0.4 (*n* = 4, mean ± SE) respectively. (C) Left: single-channel traces of mutant YnaI^M158A^ were recorded at +40 mV in different asymmetric potassium salt solutions. YnaI^M158A^ opened spontaneously without pressure applied to the pipette in asymmetric KCl, KF, and KNO_3_ solutions. Right: I–V curves for YnaI^M158A^ at different asymmetric potassium salt solutions. In KCl, KF and KNO_3_, the reversal potentials were −28.2 ± 2.6 (*n* = 6, mean ± SE), −26.5 ± 1.9 (*n* = 6, mean ± SE), −35.1 ± 0.5 (*n* = 7, mean ± SE), respectively. (D) Left: single-channel traces of mutant YnaI^Δ2−63^ were recorded at +40 mV in different asymmetric potassium salt solutions. The required negative pressures for opening the channel were different in those solutions. In KF, the required negative pressure was −60 ± 9 mmHg (*n* = 4, mean ± SE). In KCl and KNO_3_, YnaI^Δ2−63^ opened spontaneously without pressure applied (*n* = 5, *n* = 4, respectively). Right: I–V curves for YnaI^Δ2−63^ at different asymmetric potassium salt solutions. The reversal potentials of YnaI^Δ2−63^ varied in different solutions. In KF, KCl and KNO3, the reversal potentials were 14.4 ± 1.7 (*n* = 4, mean ± SE), −39.2 ± 0.6 (*n* = 5, mean ± SE), 51.9 ± 0.8 (*n* = 4, mean ± SE), respectively

The critical role of a hydrophobic residue methionine in cation selectivity has intrigued us to pursue further mechanistic insights. Although it has been observed previously that a methionine-formed circle element contributed to cation selectivity in ion channels but the mechanism has not been described clearly, such as TRP (Liao et al., 2013; Zubcevic et al., 2016), TPC (Guo et al., 2016) and Slo2.2 (Hite et al., 2015). To elucidate the nature of Na^+^/K^+^ selectivity determined by the SMC element in YnaI, we performed systematic quantum chemical investigations of the chemical bonding, charge distribution, and binding energy (Gibbs free energy, ΔG) of the protein with a series of biological relevant cations and anions (M = Na^+^, K^+^, F^−^, Cl^−^, and NO_3_^−^) (Fig. 4A and Table S1). To our surprise, the anions (F^−^, Cl^−^, and NO_3_^−^) represent much higher binding energies than that of the cations (Na^+^, ΔG = −0.87 kcal·mol^−1^ and K^+^, ΔG = 3.51 kcal·mol^−1^). Among these three anions, F^−^ has the largest binding energy (−23.31 kcal·mol^−1^) with the calculated structure elements, while NO_3_^−^ has the lowest binding energy (−13.83 kcal·mol^−1^), with Cl^−^ in the middle (−16.56 kcal·mol^−1^). Therefore, the occupation of the anions in YanI probably hampers the binding of Na^+^/K^+^ to SMC element. The data led us to hypothesize that ion binding energy could be relevant to the abilities of the anions to block cation transportation (F^−^ > Cl^−^ > NO_3_^−^) and even the ion selectivity.

Our model predicts that higher pressure would be needed to overcome the energy barrier to open the channel in KF solutions, due to the tight interaction of F^−^ with the SMC element; while in KNO_3_ solutions lesser pressure is required. To test this hypothesis, we measured the electrophysiological activities of YnaI in the solutions of K^+^ in combination with different anions using patch-clamp system and *in vitro* reconstituted giant liposomes. Indeed, in KF solutions, relatively high pressure is required to obtain detectable currents of the YnaI channel, while in KNO_3_ solutions, the YnaI channel could open spontaneously with much higher conductance even in the absence of any pressure (Fig. 4B). Surprisingly, YanI in KF solution shows a lower mean reversal potentials (−19.2 ± 1.6 mV, *n* = 4 patches), while in KNO_3_ solution displays a much negative mean reversal potentials (−52.3 ± 0.4 mV, *n* = 4 patches) in comparison with that of in KCl solution. Correspondingly, the cation-to-anion permeability ratio decreased to about 2.6:1 in KF solutions (P_K_:P_F_), while raised to about 39.6:1 in KNO_3_ solutions (P_K_:P_NO3_) (Fig. 4B and Table S2). In addition, mutant YanI^M158A^ could open spontaneously without applied any pressure with almost the same ion selectivity in above three different solutions, which implies that selectivities influenced by the anions almost vanish, further highlights the important role of SMC element in ion selectivity (Fig. 4C and Table S2). These data provide strong evidence that the ion selectivity and transmittance of YnaI could be determined by the different anions.

Previous studies demonstrated that the TM1 and TM2 may be responsible for gating the channel, and truncation of these two helixes would induce the channel to open spontaneously and present the typical gain-of-function behaviours when expressed in the living cells (Bottcher et al., 2015). Consistently, electrophysiological studies of single channel with the YnaI^Δ2−63^ mutant clearly show that the channel can open spontaneously in KCl solutions without any external pressure (Fig. S6). Apparently, the YnaI^Δ2−63^ mutant also opens spontaneously in KNO_3_ solutions. External pressure is still needed to open the truncation in KF solutions albeit at much smaller level. The ion conductance and ion selectivity of YnaI^Δ2−63^ are almost same as that of wild type YnaI in these three solutions (Fig. 4D and Table S2). These data strengthen the conclusion that TM1 and TM2 play roles in the gating of YnaI, but do not change the influence of anions on selectivity and gating manner of YnaI, which further support the critical role of YanI^M158^ and SMC in the TM region.

## Discussion

Based on the results, we proposed a new binding-block model for the molecular mechanism of how the ions were selected by the ion channels and how the mechanosensitive channels were gated by the ions. In the case of YnaI channel, different binding affinities of disparate anions to the ion selective filter (SMC) due to chemical interactions (Fig. 4A and Table S1), leads to different degree blockade of the channel gating. Among the cases we studied, the F^−^ ion has the strongest binding affinity with SMC, so the channel needs the highest pressure to overcome the energy barrier to open the channel. When the channels are forced to open at high pressure, K^+^ could pass through the SMC, and the F^−^ might move together with K^+^, causing low ion selectivity and currents in KF solutions. While in the KNO_3_ solutions, NO_3_^−^ binds to the SMC with the lowest affinity, therefore the channels could open spontaneously without pressure applied. At the same time, the lowest affinity of NO_3_^−^ binding to the SMC prevents the transportation of NO_3_^−^ which may lead to very high cation selectivity (P_K_:P_NO3_ = 39.6:1). Most important of all, as the most vital and abundant anion in living organisms, Cl^−^ has just the right binding affinity with the SMC to gate the channel, and the precise regulation has chosen methionine residues as key determination possibly after long course evolution upon high selection power.

Together, our structural, biological, biochemical, quantum mechanical, and electrophysiological results provided strong evidence that the MscS-like YnaI channel selected Na^+^/K^+^ cations specifically through the SMC element at the TM pore and the ion permeability and selectivity were determined by the anions present in the circumstances. These results not only explain why the YnaI is highly selective, but also lead to the novel binding-block mechanism of the gating and ion selectivities of ion channels.

## Materials and methods

### Protein expression and purification

Gene YnaI was cloned from *E*. *coli* into pET-21b vector (Novagen) with a C-terminal 6× His tag. Overexpression of YnaI was induced in *E*. *coli* strain BL21 (DE3) by 0.5 mmol/L isopropyl-β-D-thiogalactoside when the cell density reached OD_600_ = 1.0. After growth for 4 h at 37°C, the cells were collected, resuspended in buffer containing 20 mmol/L Tris pH 8.0, 200 mmol/L NaCl, and lysed by sonication. Cell debris was removed by centrifugation at 15,422 ×*g* for 15 min. The supernatant containing membrane was applied to ultracentrifugation at 173,021 ×*g* for 1 h. The membrane fraction was collected and incubated with 1.5% (*w*/*v*) n-dodecyl-β-D-maltopyranoside (DDM; Anatrace) for 3 h with slow stirring at 4°C. After additional ultracentrifugation at 173,021 ×*g* for 30 min, the supernatant was collected and loaded onto Ni^2+^-nitrilotriacetate affinity resin (Ni-NTA; Qiagen). The resin was then washed with buffer A containing 25 mmol/L Tris pH 8.0, 20 mmol/L imidazole, 500 mmol/L NaCl, and 0.02% DDM. Followed by eluted from affinity resin with buffer A supplemented with 300 mmol/L imidazole, the protein was concentrated and applied to a gel-filtration resin (Superdex-200 HR 10/30; GE Healthcare), previously equilibrated with buffer containing 20 mmol/L Mes pH 6.5, 200 mmol/L NaCl, 5 mmol/L DTT (Dithiothreitol), and 0.02% DDM. The peak fractions were collected for cryo-EM and electrophysiology studies. Various YnaI mutants followed the same procedures.

For cryo-EM study, the protein was mixed with amphipols (Anatrace) at 1:3 (*w*/*w*) for 5 h with slow stirring at 4°C. Detergent was removed with Bio-Beads SM-2. After separation from Bio-beads, the protein was loaded to Superdex 200 again with buffer containing 20 mmol/L Mes pH 6.5, 200 mmol/L NaCl, 5 mmol/L DTT. The peak fractions were collected for analysis by cryo-EM.

### Preparation of giant liposomes and electrical recording

All lipids used in reconstitution were purchased from Avanti Polar Lipids. The wild-type YnaI and the mutant proteins were reconstituted into lipid vesicles composed of 1-palmitoyl-2-oleoyl-phosphatidylethanolamine (POPE, 7.5 mg/mL) and 1-palmitoyl-2-oleoyl-phosphatidylglycerol (POPG, 2.5 mg/mL) as previously described method (Li et al., 2007). The giant liposomes were obtained by regular dehydrate and hydrate processes. The patch-clamp recording of YnaI were performed in asymmetrical conditions with 15 mmol/L KCl or NaCl, 500 mmol/L sucrose, 5 mmol/L K-Hepes (pH 7.0) in bath solution, and 150 mmol/L KCl or NaCl, 500 mmol/L sucrose, 5 mmol/L K-Hepes (pH 7.0) in pipette solution. YnaI and YnaI ^Δ2−62^ were performed in asymmetrical KCl, KF and KNO_3_ solutions, but other mutants were performed only in asymmetrical KCl solution. Concentrations of KF and KNO_3_ used in asymmetrical conditions are same as KCl. After attained a gigohm seal (the resistance was about 3–8 GΩ), the current was recorded by using an Axopatch 200B amplifier with a Digidata 1322A analogue-to-digital converter (Axon Instruments). The mechanical pressure was measured by a pressure monitor (PM015D, WPI). Permeability ratios were calculated by using Nernst equation as following:

$$ E_{\text{rev}} = \frac{RT}{F}\ln \left( {\frac{{P_{\text{K}} [{\text{K}}]_{\text{o}} + P_{\text{Cl}} [{\text{Cl}}]_{\text{i}} }}{{P_{\text{K}} [{\text{K}}]_{\text{i}} + P_{\text{Cl}} [{\text{Cl}}]_{\text{o}} }}} \right) $$

where [X]_o_ and [X]_i_ are ion concentration on extracellular (cis-side) and intracellular sides (trans-side), respectively.

### EM sample preparation and data collection

The homogeneity of purified YnaI in amphipols were examined by negative staining with 2% uranyl acetate. Images were recorded using a 4 k × 4 k CCD camera (UltraScan 4000, Gatan) in an FEI T12 microscope operated at 120 kV. For cryo-grid preparation, the Quantifoil 1.2/1.3 holy carbon grids were baked at 50°C in an oven for 2 weeks. This treatment of grids would allow better distribution of particles into the carbon holes. 4 μL aliquots of freshly purified sample (0.1 mg/mL) were loaded on the pretreated and glow-discharged grids. Blotting and sample freezing were performed in an FEI Mark IV Vitrobot (4°C and 85% humidity). Images were recorded using a K2 Summit direct electron detector (Gatan) in super resolution counting mode at a nominal magnification of 22,500× (rendering a pixel size of 1.32 Å) on an FEI Titan Krios electron microscope at 300 kV. All images were collected by UCSF-image4 (X. Li and Y. Cheng, UCSF) with defocus ranging from −1 ~ −2.2 µm. The total exposure time is 8 s (32 frames), with a dose rate of ~8.2 counts per physical pixel per second.

### Image processing and analyses

The initial model was calculated using EMAN2 (Ludtke et al., 1999) with the 2D class averages from negatively stained particles. For cryo-EM data, the motion correction was performed with MOTIONCORR (Li et al., 2013) at micrograph level, and the estimation of contrast transfer function parameters was performed with CTFFIND3 (Mindell and Grigorieff, 2003). Micrograph screening, automatic particle picking, and particle normalization were performed with SPIDER (Shaikh et al., 2008) software packages. The 2D classification, 3D classification and refinement were performed with RELION (Scheres, 2012). A total of 550,000 particles (window size 144 × 144) were automatically picked from 2,100 micrographs. Based on the results of 2D classification, 90% of the particles were grouped into top view classes, 8% of the particles were in tilted views, and less than 2% of the particles were in standard side views. Although the tilted- and side-view particles (42,000 particles) only contribute to a minor portion of the total particles, they are essential in determining correct reconstruction. We have tried to mix different numbers of top-view particles with these tilted and side-view particles in several rounds of 3D refinement, and we found that if we omitted all top-view particles, we could get a better map in both the nominal resolution and map quality. Therefore, after region-based 3D classification and refinement, a final data set of 42,000 nontop-view particles resulted in a 3.8 Å map (gold-standard FSC 0.143). The density of the transmembrane region was relatively weak, and could not be improved after global or local 3D classification. To improve the resolution in the region of the intracellular domain, we added a soft mask around the intracellular domain in the refinement and obtained a 3.6 Å map. To further improve the map quality, we used the dose-reduced particles summed from frames of 3–18, and the overall resolution of the map has been improved to 3.6 Å. The local resolution maps were calculated by ResMap (Kucukelbir et al., 2014).

### Model building and refinement

The homologue crystal structure of *Thermoanaerobacter tengcongensis* MscS (PDB 3UDC) was docked into the density map of YnaI heptamer as a start model in Chimera (Pettersen et al., 2004). Sequence alignment of YnaI with the crystal template was performed by BLAST (Mount, 2007). The atomic model of the intracellular domain was manually built in coot (Emsley et al., 2010) based on the start model with the Mutate and Renumber tools. Sequence assignment was mainly guided by the clearly resolved bulky residues (such as Phe, Tyr, Trp, and Arg). This model was refined by real-space refinement (phenix.real_space_refine) in Phenix (Adams et al., 2010), with stereochemical and secondary structure constraints applied. The refined model was examined by cross-validation according to previously described procedures (Li et al., 2015). Specifically, the coordinates of the refined model were randomly shifted by 0.2 Å using Phenix PDB tool. The shifted model was then refined with half1 map in Phenix. The new refined model (with half1 map) was converted to mrc map, and then compared with half1 map, half2 map and combined map to calculate the FSC curves, respectively. These curves indicated that the model was not overfitted. The model of transmembrane domain was built in MDFF (Trabuco et al., 2009). We deleted all the side chains in this region due to the resolution limitation.

### Quantum chemical methods and computational details

The geometries of the model systems (H_3_CSCH_3_)_7_ and A@(H_3_CSCH_3_)_7_ (A = Na^+^, K^+^, F^−^, Cl^−^, NO_3_^−^) were optimized via constrained energy minimization at the level of density functional theory (DFT). All the atomic coordinates are optimized except the positions of the inner ring formed by C atoms and all the S atoms that were fixed at the experimental structures. The B3LYP hybrid exchange-correlation function (Becke, 1993; Lee et al., 1988) was used with 6–31 + G* basis sets (Francl et al., 1982) for all the elements. The vibrational frequencies were calculated with the harmonic approximation. The thermodynamic properties were calculated at ambient temperature and pressure using statistical mechanics. All the calculations were carried out by using Gaussian 09 program (Frisch, M. J. et al. Gaussian 09. Revision C.01 (Gaussian, 2010).)

## Electronic supplementary material

Below is the link to the electronic supplementary material.

Supplementary material 1 (PDF 1135 kb)
