# Correction to: A binding-block ion selective mechanism revealed by a Na/K selective channel

**DOI:** 10.1007/s13238-019-0619-y

**Published:** 2019-03-25

**Authors:** Jie Yu, Bing Zhang, Yixiao Zhang, Cong-qiao Xu, Wei Zhuo, Jingpeng Ge, Jun Li, Ning Gao, Yang Li, Maojun Yang

**Affiliations:** 1 Ministry of Education Key Laboratory of Protein Science, School of Life Sciences, Tsinghua-Peking Joint Center for Life Sciences, Beijing Advanced Innovation Center for Structural Biology, Tsinghua University, Beijing, 100084 China; 2 Key Laboratory of Receptor Research, Shanghai Institute of Materia Medica, Chinese Academy of Sciences, Shanghai, 201203 China; 3 Department of Chemistry and Key Laboratory of Organic Optoelectronics and Molecular Engineering of the Ministry of Education, Tsinghua University, Beijing, 100084 China; 4 University of Chinese Academy of Sciences, Beijing, 100049 China; 5 Anesthesiology, Shanghai First Maternity and Infant Hospital, Tongji University school of Medicine, Shanghai, 201203 China

## Correction to: Protein Cell 2018, 9(7):629–639 10.1007/s13238-017-0465-8

In the original publication the PDB numbers were not cited.

The correct version is provided in this correction article.

Three-Dimensional ctyo-EM density maps and coordinates of YnaI has been deposited in the Electron Microscopy Data Bank under the accession number EMD-6805 and deposited in the RCSB Protein Data Bank under the accession code 5Y4O.

