# Supplementary material for: A binding-block ion selective mechanism revealed by a Na/K selective channel
Source: Protein Cell. 2017 Sep 18;9(7):629–39. doi: 10.1007/s13238-017-0465-8 (PMC6019658; doi:10.1007/s13238-017-0465-8)
Supplement: Supplementary file 1 — Supplementary material 1 (PDF 1135 kb) [file 13238_2017_465_MOESM1_ESM.pdf]

Supporting information

Supplementary Figures

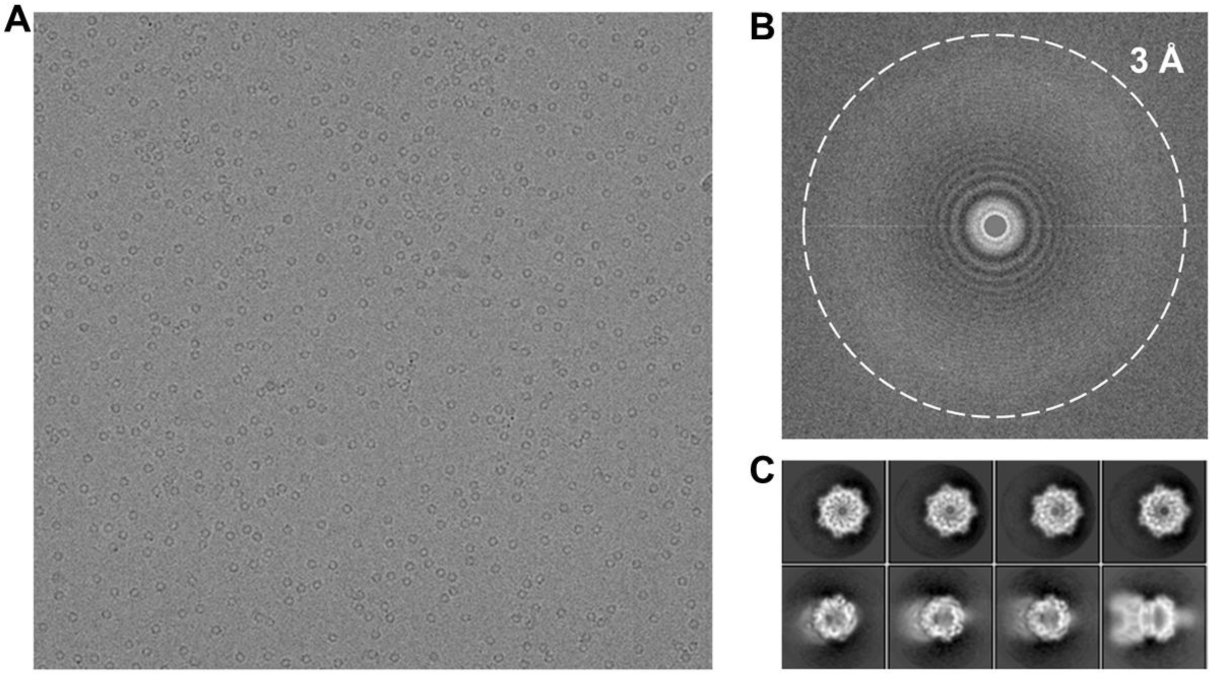

Fig. S1. A representative micrograph collected with Titan Krios and K2 camera

A. A representative YnaI Cryo-EM image after motion correction. The particles show a strong preferred orientation.

B. Fourier transform of the image shown in (A). The dash cycle indicates the 3Å resolution in the Thon ring.

C. The 2D class averages of the Cryo-EM dataset. The upper row shows the top view classes and the lower row shows the tilt and side view classes.

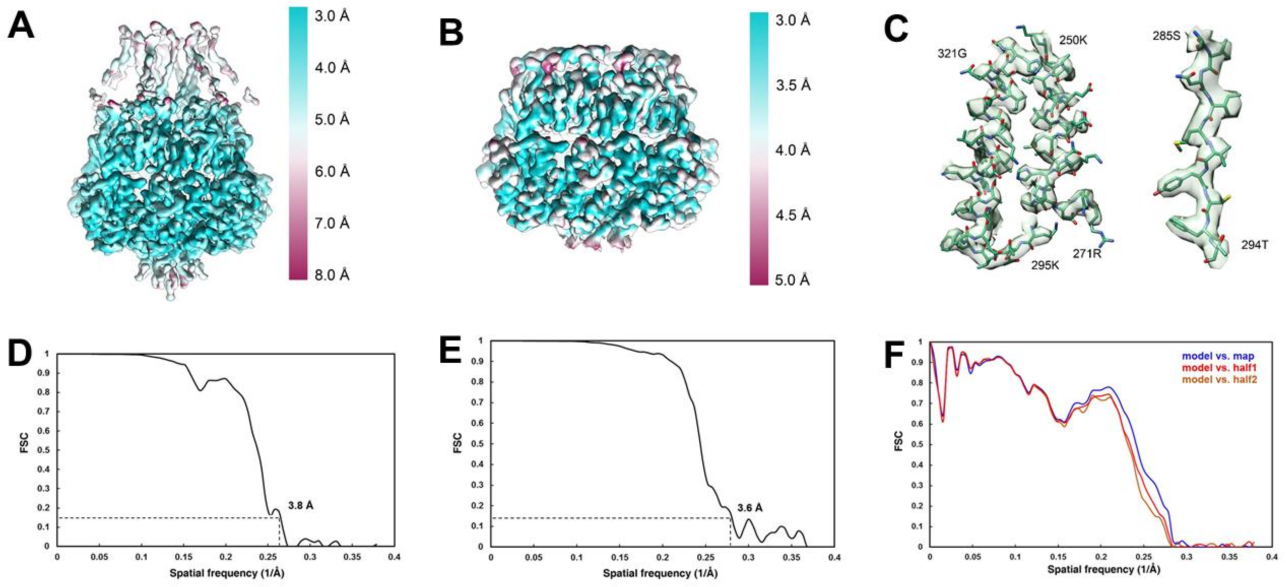

Fig. S2. Local resolution maps and FSC curves

A-B. The local resolution map of the overall structure (a) and the intracellular domain structure (b) refined within a soft mask.

C. The Cryo-EM densities of the selected region were superimposed with the atomic model.

D-E. The FSC curve of the overall refinement (a) and the intracellular domain refinement (b) within a soft mask.

F. The FSC curve of the intracellular domain atomic model cross-validation.

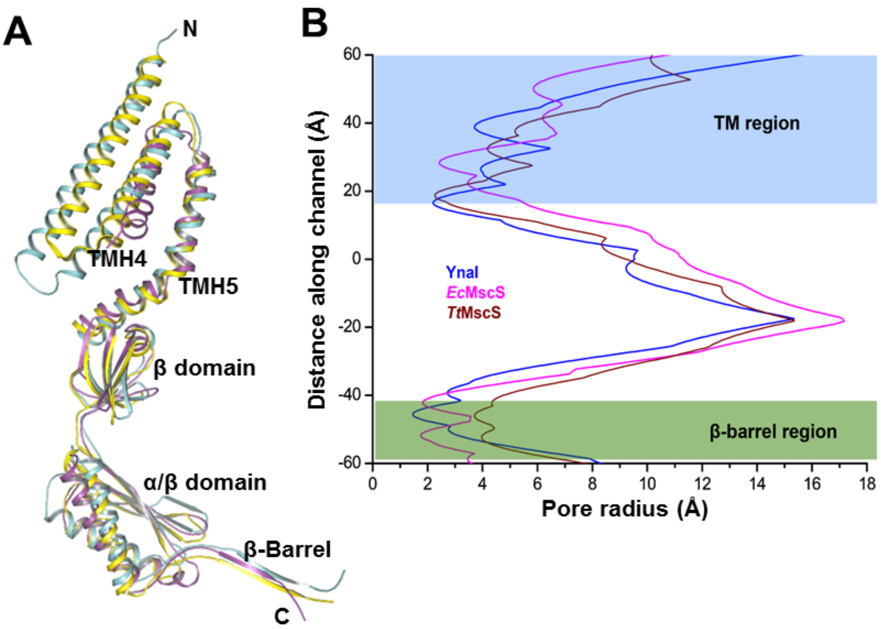

Fig. S3. Overview structure of YnaI in the closed conformation

A. YnaI is in a closed conformation. Stereo views of structural alignment of one single YnaI with *Tt*MscS (Protein Data Bank code: 3UDC) and closed state of *Ec*MscS (Protein Data bank code: 2OAU). Protomers of YnaI, *Tt*MscS and *Ec*MscS are shown in purple, cyan and yellow, respectively.

B. Plots of pore radiuses of YnaI, *Tt*MscS and *Ec*MscS, shown in blue, magenta and brown colors, respectively.

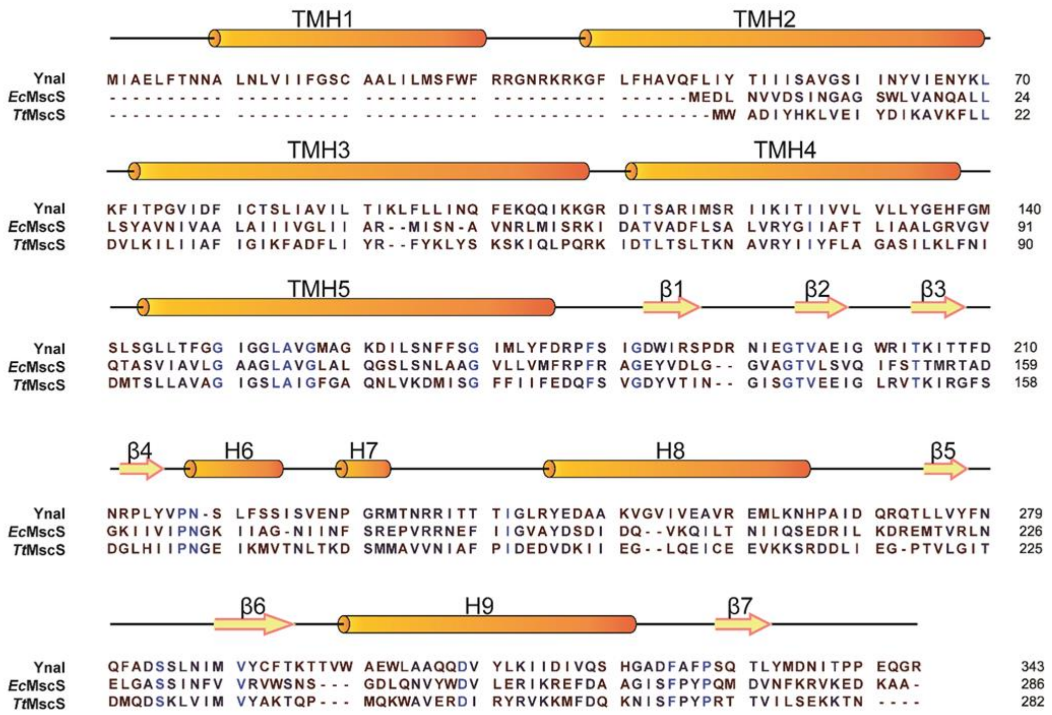

Fig. S4. YnaI contains five TMHs

Sequence alignment of YnaI, *Tt*MscS and *Ec*MscS. Conserved residues are highlighted in blue. Secondary structural elements of YnaI are indicated above the sequence.

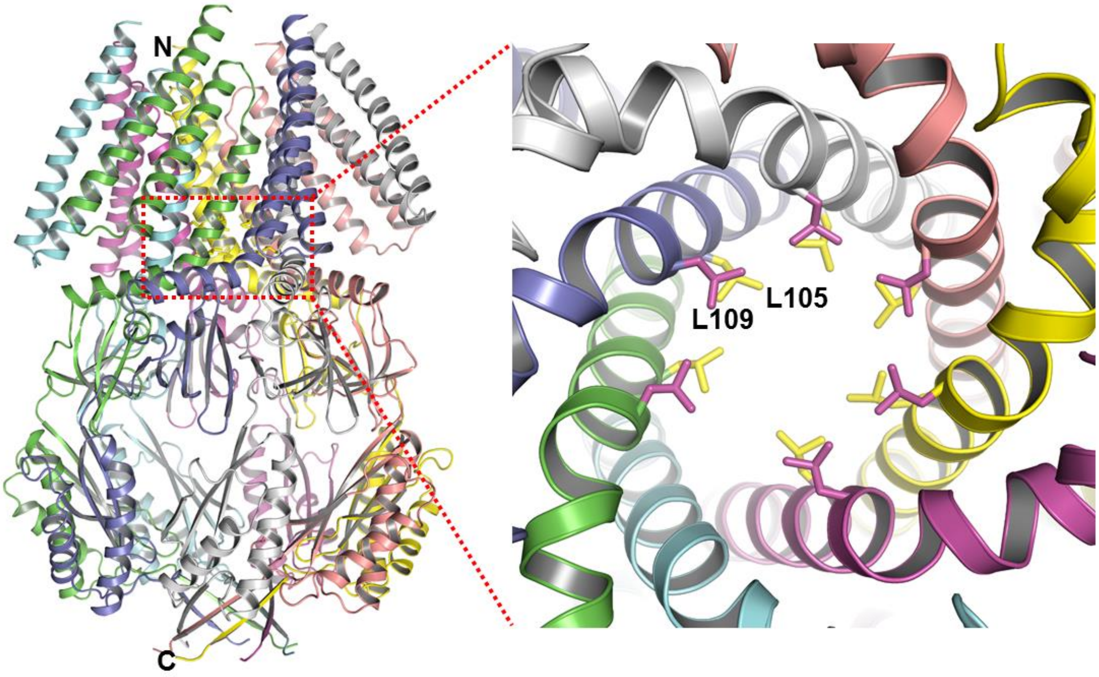

Fig. S5. The *Ec*MscS$^{L105\ and\ L109}$ residues gate the channel

Left: Cartoon represents the overall structure of *Ec*MscS homoheptamer. Different subunits are shown in different colors.

Right: The *Ec*MscS$^{L105\ and\ L109}$ residues locate at the gating site of this channel as indicated in left panel. The *Ec*MscS^{L105} and *Ec*MscS^{L109} residues are shown in yellow and magenta sticks and indicated, respectively.

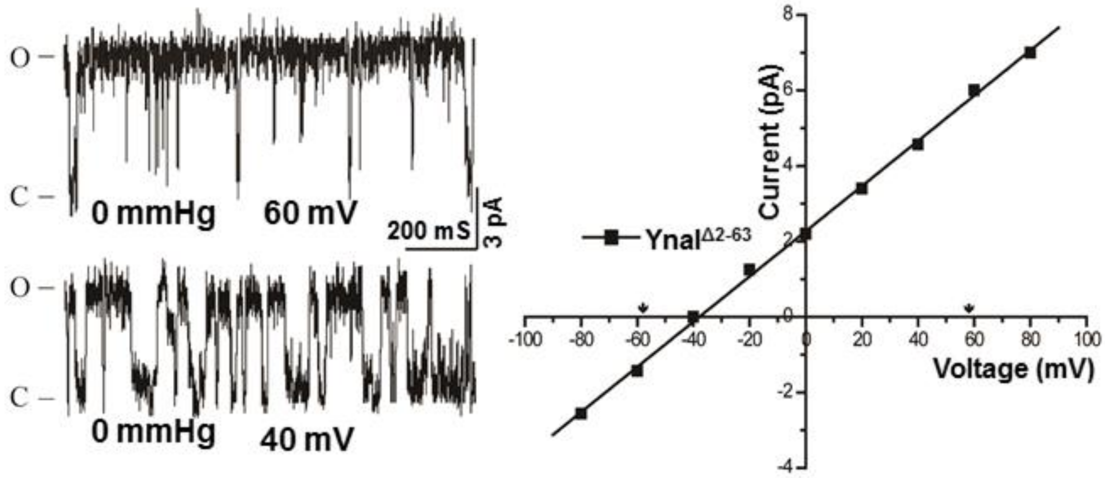

Fig. S6. TM1 and TM2 of YnaI are responsible for gating the channel

Left: spontaneous current traces recorded at +40 mv and +60 mv from the YnaI$^{\Delta 2\text{-}63}$ mutant without application of pressure. Right: I-V curves for the YnaI$^{\Delta 2\text{-}63}$. The reversal potential measured from the I-V curve was −39.2 ± 0.6 mV (n = 5, mean ± SE).

Supplementary Tables

Table S1. Calculated quantum chemical and thermodynamic energies for the process A + $(H_3CSCH_3)_7 \rightarrow A@(H_3CSCH_3)_7$ (A = Na^+, K^+, F^-, Cl^-, NO_3^-)

Ion types (A)	ΔE_e/kcal·mol^{-1}	ΔE_0/kcal·mol^{-1}	ΔH/kcal·mol^{-1}	ΔG/kcal·mol^{-1}
Na^+	-8.65	-8.85	-9.09	-0.87
K^+	-2.50	-3.17	-3.09	3.51
F^-	-32.59	-31.78	-32.24	-23.31
Cl^-	-22.32	-22.02	-21.90	-16.56
NO_3^-	-23.50	-22.54	-21.85	-13.83

* E_e and E_0 denote the calculated quantum mechanical energies at the equilibrium structures and the ones corrected with zero-point energies. H and G are the enthalpies and Gibbs free energies calculated at 298 K and 101.325 kPa (1 atm).

Table S2. Property summary of YnaI, YnaI^{M158A} and YnaI$^{\Delta 2\text{-}63}$ in different solutions in this study

Proteins	Solvents	Vrev (mV)	Px/Py	Conductance (r, pS)	n
**YnaI**	**KCl**	**-39.2±0.5**	**8.9±0.3**	**43±0.34**	**5**
	**KF**	**-19.2±1.6**	**2.6±0.2**	**66±6.12**	**4**
	**KNO_3**	**-52.3±0.4**	**39.6±5.0**	**216±16.6**	**4**
**YnaI^{M158A}**	**KCl**	**-26.5±1.9**	**4±0.5**	**108.6±29.7**	**6**
	**KF**	**-28.2±2.6**	**4.5±0.7**	**135.3±44.7**	**6**
	**KNO_3**	**-35.1±0.5**	**6.6±0.2**	**99.3±12.5**	**7**
**YnaI $^{\Delta 2\text{-}63}$**	**KCl**	**-39.2±0.6**	**9.0±0.5**	**82.9±2.98**	**5**
	**KF**	**-14.4±1.7**	**2.0±0.2**	**52.2±2.2**	**4**
	**KNO_3**	**-51.9±0.8**	**39.3±4.7**	**156.3±16.23**	**4**

The reversal potential (Vrev) and conductance were measured through the I-V curve. The applied negative pressures were monitored by piezoelectric pressure transducer (World Precision Instruments).
